# Supplementary material for: The Q223R Polymorphism of the Leptin Receptor Gene as a Predictor of Weight Gain in Childhood Obesity and the Identification of Possible Factors Involved
Source: Genes (Basel). 2020 May 17;11(5):560. doi: 10.3390/genes11050560 (PMC7288327; doi:10.3390/genes11050560)
Supplement: Supplementary file 1 [file genes-11-00560-s001.pdf]

Supplementary Information.

Supplementary Table: Associations results between the 11 SNPs studied and anthropometric annual change variables in the codominant linear models adjusted by sex and age.

SNP	CH [(1)]	Het [(1)]	VH [(1)]	Beta [(2)]	P.Val(*)	P.Val.Adj (**)
**Weight growth (kg/year)**						
rs17782313	3.2 ± 1.79	3.28 ± 1.68	3.36 ± 1.84	0.08 (-0.48-0.65) / 0.28 (-1.29-1.86)	0.909	1
rs6548238	3.33 ± 1.76	3.02 ± 1.71	3.29 ± 1.95	-0.31 (-0.85-0.24) / 0.05 (-0.99-1.09)	0.523	1
rs10938397	3.29 ± 1.69	3.24 ± 1.91	3.11 ± 1.53	-0.12 (-0.7-0.46) / -0.17 (-0.85-0.51)	0.874	1
rs368794	3.02 ± 1.83	3.36 ± 1.69	3.42 ± 1.70	0.31 (-0.22-0.83) / 0.43 (-0.35-1.22)	0.391	1
rs2568958	3.31 ± 1.59	3.08 ± 1.79	3.43 ± 2.06	-0.22 (-0.75-0.31) / 0.12 (-0.62-0.87)	0.567	1
rs925946	3.13 ± 1.79	3.50 ± 1.70	2.60 ± 1.59	0.35 (-0.17-0.87) / -0.51 (-1.48-0.46)	0.167	1
rs7647305	3.19 ± 1.70	3.34 ± 1.90	3.05 ± 1.76	0.18 (-0.37-0.73) / -0.04 (-1.48-1.39)	0.804	1
rs10913469	3.07 ± 1.64	3.63 ± 1.80	3.30 ± 2.83	0.48 (-0.08-1.04) / 0.23 (-0.95-1.41)	0.241	1
rs7190492	3.21 ± 1.69	3.18 ± 1.75	3.45 ± 1.98	-0.07 (-0.62-0.47) / 0.14 (-0.61-0.88)	0.852	1
rs7903146	3.41 ± 1.83	3.28 ± 1.78	2.53 ± 1.24	-0.02 (-0.55-0.51) / -0.75 (-1.53-0.02)	0.130	1
rs1137101	3.52 ± 1.79	2.80 ± 1.49	3.99 ± 2.08	-0.72 (-1.26--0.18) / 0.42 (-0.3-1.13)	0.001	0.012
**Height growth (cm/year)**						
rs17782313	5.73 ± 1.02	5.60 ± 0.93	5.98 ± 0.94	-0.12 (-0.44-0.2) / 0.26 (-0.63-1.15)	0.608	1
rs6548238	5.81 ± 0.97	5.47 ± 1.01	5.61 ± 1.00	-0.35 (-0.65--0.04) / -0.28 (-0.87-0.3)	0.070	0.774
rs10938397	5.75 ± 1.04	5.69 ± 1.00	5.63 ± 0.91	-0.06 (-0.39-0.26) / -0.17 (-0.55-0.22)	0.690	1
rs368794	5.69 ± 0.93	5.80 ± 1.05	5.36 ± 0.91	0.12 (-0.17-0.41) / -0.42 (-0.85-0.02)	0.053	0.586
rs2568958	5.63 ± 1.07	5.75 ± 0.87	5.72 ± 1.12	0.12 (-0.18-0.42) / 0.1 (-0.32-0.52)	0.729	1
rs925946	5.65 ± 0.97	5.80 ± 1.,4	5.56 ± 0.86	0.15 (-0.14-0.44) / -0.09 (-0.64-0.46)	0.519	1
rs7647305	5.69 ± 1.03	5.71 ± 0.87	5.73 ± 1.13	-0.01 (-0.32-0.3) / -0.04 (-0.86-0.77)	0.992	1
rs10913469	5.63 ± 0.90	5.74 ± 1.08	6.39 ± 1.51	0.16 (-0.16-0.47) / 0.81 (0.15-1.47)	0.045	0.498
rs7190492	5.69 ± 0.95	5.68 ± 1.06	5.78 ± 0.94	-0.03 (-0.33-0.28) / 0.1 (-0.32-0.52)	0.840	1
rs7903146	5.76 ± 1.00	5.73 ± 0.95	5.37 ± 1.09	-0.09 (-0.39-0.21) / -0.48 (-0.91--0.04)	0.101	1
rs1137101	5.77 ±0.85	5.56 ± 1.06	5.94 ± 1.00	-0.21 (-0.52-0.1) / 0.23 (-0.18-0.64)	0.063	0.697

**BMI variation (unitless)**						
rs17782313	2.21 ± 4.48	3.16 ±5.21	2.83 ± 5.29	0.96 (-0.56-2.49) / 1.02 (-3.21-5.26)	0.434	1
rs6548238	2.53 ± 4.60	2.34 ± 4.64	3.03 ± 6.31	-0.21 (-1.72-1.29) / 0.55 (-2.28-3.37)	0.875	1
rs10938397	2.24 ± 4.54	2.51 ± 4.82	2.80 ± 4.72	0.11 (-1.48-1.69) / 0.52 (-1.34-2.39)	0.843	1
rs368794	1.69 ± 4.91	2.71 ± 4.33	4.49 ± 4.73	0.94 (-0.46-2.35) / 2.73 (0.58-4.88)	0.041	0.452
rs2568958	2.82 ± 4.35	2.02 ± 4.84	3.10 ± 5.18	-0.8 (-2.24-0.64) / 0.29 (-1.75-2.32)	0.421	1
rs925946	2.37 ± 4.81	3.03 ± 4.54	0.91 ± 4.55	0.55 (-0.87-1.97) / -1.4 (-4.03-1.23)	0.345	1
rs7647305	2.29 ± 4.41	3.01 ± 5.22	2.42 ± 6.27	0.71 (-0.8-2.21) / 0.19 (-3.69-4.07)	0.651	1
rs10913469	2.24 ± 4.55	3.30 ± 4.81	1.94 ± 6.22	0.96 (-0.57-2.49) / -0.18 (-3.38-3.02)	0.448	1
rs7190492	2.38 ± 4.49	2.63 ± 4.61	2.56 ± 5.60	0.06 (-1.43-1.55) / -0.11 (-2.12-1.91)	0.987	1
rs7903146	3.08 ± 4.63	2.43 ± 4.97	1.06 ± 3.86	-0.44 (-1.9-1.03) / -1.82 (-3.93-0.3)	0.24	1
rs1137101	3.36 ± 5.04	1.63 ± 4.30	3.63 ± 4.87	-1.73 (-3.23--0.23) / 0.24 (-1.76-2.23)	0.027	0.294
**Fat mass variation (unitless)**						
rs17782313	0.16 ± 13.13	2.8 ± 15.00	1.15 ± 20.41	2.59 (-1.9-7.08) / 0.23 (-12.2-12.65)	0.523	1
rs6548238	1.25 ± 13.75	1.1 ± 13.9	-0.56± 16.34	-0.15 (-4.59-4.3) / -0.61 (-8.92-7.69)	0.989	1
rs10938397	-0.32 ± 11.57	0.6 ± 13.66	3.48± 16.74	1.46 (-3.19-6.11) / 4.71 (-0.75-10.17)	0.225	1
rs368794	-1.23 ± 15.64	2.36 ± 12.34	3.88± 12.01	3.6 (-0.57-7.78) / 6.6 (0.25-12.96)	0.071	0.786
rs2568958	2.00 ± 13.21	-0.74± 14.6	3.68± 13.28	-2.58 (-6.83-1.67) / 1.62 (-4.36-7.6)	0.281	1
rs925946	0.88±14.69	2.15±13.02	-3.05± 11.79	1.62 (-2.56-5.8) / -4.02 (-11.99-3.96)	0.374	1
rs7647305	1.30±14.25	0.88±12.21	-4.17± 21.06	0.18 (-4.26-4.61) / -4.15 (-15.57-7.28)	0.764	1
rs10913469	0.68± 14.34	2.99± 13.19	-4.01± 10.12	1.82 (-2.66-6.31) / -5.56 (-14.94-3.82)	0.316	1
rs7190492	-0.01± 11.83	3.1± 15.44	-1.11± 14.63	3.91 (-0.45-8.28) / -0.45 (-6.3-5.4)	0.142	1
rs7903146	2.37± 12.57	1.13± 14.65	-2.6± 15.18	-1.07 (-5.41-3.27) / -4.34 (-10.58-1.89)	0.39	1
rs1137101	2.37± 13.71	0.00± 14.72	1.91± 11.76	-2.21 (-6.72-2.3) / -1.09 (-7.06-4.89)	0.623	1
**Muscle mass variation (unitless)**						
rs17782313	7.49 ± 5.09	7.18 ± 5.12	5.49 ± 4.19	-0.17 (-1.67-1.34) / -1.4 (-5.35-2.54)	0.774	1
rs6548238	7.44 ± 5.12	6.68 ± 5.01	10.0 1± 3.86	-0.77 (-2.24-0.7) / 1.43 (-1.91-4.78)	0.361	1
rs10938397	6.68 ± 5.86	7.61 ± 4.51	7.64 ± 5.00	0.54 (-1.02-2.1) / 0.18 (-1.69-2.04)	0.777	1
rs368794	6.96 ± 5.06	7.58 ± 5.37	7.74 ± 3.61	0.46 (-0.97-1.89) / -0.37 (-2.57-1.83)	0.684	1
rs2568958	6.51 ± 4.41	8.08 ± 5.34	7.32 ± 5.54	1.33 (-0.11-2.76) / 0.8 (-1.2-2.79)	0.191	1
rs925946	7.35 ± 5.19	7.29 ± 5.06	7.26 ± 4.3	-0.32 (-1.75-1.11) / -0.16 (-2.8-2.48)	0.907	1
rs7647305	7.31 ± 5.26	7.45 ± 4.68	6.98 ± 4.87	-0.83 (-2.3-0.63) / -2.27 (-5.87-1.33)	0.296	1
rs10913469	7.85 ± 5.45	6.40 ± 4.08	5.59 ± 3.64	-0.84 (-2.34-0.67) / -0.95 (-4.11-2.21)	0.499	1
rs7190492	6.82 ± 4.86	8.02 ± 5.53	6.89 ± 4.12	0.23 (-1.28-1.73) / -0.7 (-2.73-1.33)	0.66	1

rs7903146	7.72 ± 5.27	6.95 ± 5.15	6.83 ± 3.45	-1.21 (-2.66-0.24) / -1.97 (-4.07-0.14)	0.109	1
rs1137101	6.81 ± 4.87	7.74 ± 5.05	6.76 ± 5.04	0.62 (-0.92-2.15) / 0.51 (-1.43-2.45)	0.723	1
**Waist circumference variation (unitless)**						
rs17782313	2.44 ± 4.47	2.76 ± 3.35	5.10 ± 5.34	0.34 (-1.03-1.7) / 2.91 (-0.9-6.73)	0.306	1
rs6548238	2.71 ± 4.13	2.48 ± 4.20	2.10 ± 5.50	-0.23 (-1.56-1.11) / -0.53 (-3.08-2.01)	0.886	1
rs10938397	2.31 ± 4.31	2.54 ± 3.98	3.05 ± 4.60	0.13 (-1.29-1.55) / 0.76 (-0.91-2.42)	0.631	1
rs368794	2.22 ± 4.48	2.69 ± 3.89	3.43 ± 4.42	0.43 (-0.86-1.71) / 1.25 (-0.66-3.15)	0.427	1
rs2568958	2.92 ± 4.36	2.34 ± 3.90	2.47 ± 4.79	-0.57 (-1.87-0.73) / -0.43 (-2.24-1.38)	0.679	1
rs925946	2.59 ± 4.17	2.86 ± 4.41	1.36 ± 3.56	0.22 (-1.05-1.49) / -1.19 (-3.56-1.18)	0.521	1
rs7647305	2.33 ± 3.95	3.20 ± 4.83	2.62 ± 3.69	0.91 (-0.42-2.24) / 0.41 (-3.08-3.89)	0.404	1
rs10913469	2.28 ± 4.02	3.45 ± 4.51	2.50 ± 5.24	1.07 (-0.3-2.44) / 0.27 (-2.61-3.14)	0.307	1
rs7190492	2.66 ± 4.18	2.40 ± 4.18	2.97 ± 4.55	-0.37 (-1.7-0.96) / 0.12 (-1.69-1.93)	0.807	1
rs7903146	3.12 ± 4.52	2.49 ± 4.28	1.43 ± 2.70	-0.48 (-1.8-0.83) / -1.53 (-3.43-0.37)	0.281	1
rs1137101	3.80 ± 4.35	1.51 ± 3.91	3.70 ± 4.13	-2.3 (-3.61--0.99) / -0.16 (-1.91-1.6)	9.11e-04	0.010
**Triceps fold variation (unitless)**						
rs17782313	0.23 ± 15.79	0.97 ± 16.52	4.33 ± 19.34	0.88 (-4.36-6.11) / 5.2 (-9.32-19.72)	0.753	1
rs6548238	0.20 ± 14.22	0.38 ± 16.85	3.97 ± 27.41	0.07 (-5.01-5.16) / 3.13 (-6.54-12.8)	0.813	1
rs10938397	-0.07 ± 11.83	0.38 ± 17.71	1.32 ± 17.25	-0.12 (-5.52-5.28) / 0.83 (-5.52-7.17)	0.947	1
rs368794	-0.27 ± 18.12	0.92 ± 14.45	1.2 ± 14.21	1.07 (-3.84-5.98) / 0.52 (-6.77-7.8)	0.912	1
rs2568958	2.83 ± 14.83	-1.12 ± 16.49	-1.19 ± 17.18	-4.05 (-8.95-0.86) / -3.85 (-10.78-3.08)	0.233	1
rs925946	0.01 ± 16.67	2.12 ± 15.25	-4.7 ± 14.42	1.86 (-2.96-6.68) / -4.39 (-13.69-4.91)	0.406	1
rs7647305	0.64 ± 15.21	0.8 ± 17.91	-6.37 ± 14.25	-0.28 (-5.36-4.8) / -7.81 (-21.06-5.43)	0.509	1
rs10913469	-0.82 ± 15.43	3.73 ± 16.21	0.88 ± 21.97	4.73 (-0.47-9.93) / 2.42 (-8.46-13.31)	0.199	1
rs7190492	0.25 ± 13.37	0.36 ± 16.66	1.54 ± 21.02	-0.56 (-5.63-4.5) / 0.6 (-6.34-7.54)	0.941	1
rs7903146	2.52 ± 17.07	-0.13 ± 15.32	-3.69 ± 14.44	-2.56 (-7.53-2.42) / -6.42 (-13.61-0.76)	0.199	1
rs1137101	2.76 ± 17.55	-1.93 ± 15.68	3.57 ± 12.86	-4.78 (-9.88-0.33) / 1.17 (-5.64-7.99)	0.075	0.823

CH, Common Homocygote; Het, Heterocygote; VH, Variant Hommocygote

(1) Mean ± Sd

(2) Co-dominant genetic model (AA vs Aa / AA vs aa)

(*) P.val of SNP adjusted by age and sex

(**) Bonferroni-corrected p.value

SNPs selection (Major allele/minor allele): rs17782313 (T/C); rs6548238 (C/T); rs10938397 (A/G); rs368794 (A/T); rs2568958 (A/G); rs925946 (G/T); rs7647305 (C/T); rs10913469 (T/C); rs7190492 (G/A); rs7903146 (C/T); rs1137101 (A/G).
